# The Ethnographic Interview: An Interdisciplinary Guide for Developing an Ethnographic Disposition in Health Research

**DOI:** 10.1177/10497323241241225

**Published:** 2024-08-07

**Authors:** Catherine Trundle, John Gardner, Tarryn Phillips

**Affiliations:** 1 2080 La Trobe University, Melbourne, VIC, Australia; 2 2541 Monash University, Melbourne, VIC, Australia

**Keywords:** ethnographic interview, ethnographic disposition, interviews, methodology, qualitative interview, health ethnography

## Abstract

Interviews are central to the health ethnographers’ toolkit. In this article, we offer a critical engagement with methodological literature coupled with reflective examples from our own research, in order to articulate the value of the ethnographic interview in health research. We contribute to literature on ethnographic interviews in two ways: by decoupling ethnographic interviews from the necessity of accompanying participant observation, and by outlining an ethnographic disposition towards interviewing. We define the seven key epistemic dispositions underpinning the ethnographic interview. These are humility, a readiness to revise core assumptions about a research topic, attentiveness to context, relationality, openness to complexity, an attention to ethnographic writing, and a consideration of the politics and history of the method. The strength of an epistemic understanding of the ethnographic interview is that it offers flexibility for developing a diverse array of interview techniques responsive to the needs of different research contexts and challenges. Ethnographic interviews, we show, contribute to the study of health through a richly explorative, responsive, contextualised, and reflexive approach.

## Introduction

Interviewing is an elemental aspect of ethnographic research. Ethnographic approaches to research emerged first within anthropology and sociology in the first half of the 20th century and have become popular in a range of fields, including health sciences, nursing, education, computer science, and design (Leder Mackley & Pink, 2013; Rosenberg, 2001). Within the health sciences, ethnography is increasingly utilised to help address wide ranging health problems and has been adapted to fit the realities and demands of health research teams, medical settings, and research funding models (Cook, 2005; Kian & Beach, 2019).

What makes an interview *ethnographic*? To answer this question, this article offers a critical literature review, illustrated by personal reflective vignettes drawing upon our collective 40-plus years’ experience in conducting anthropological, sociological, and focused ethnographic interviews to study health. Rather than offer a step-by-step prescriptive guide to interviewing, we propose a set of epistemological dispositions that underpin an ethnographic approach to interviewing. This approach interrogates the deeper assumptions, goals, and rationales of ethnography. This avoids prescribing data collection approaches and allows for the flexible continued innovation of a diversity of ethnographic interviewing techniques.

## What Is Ethnography?

As Hammersley (2006) notes, there is not a singular definition of ethnography. Nor are the boundaries clear-cut between ethnography and other qualitative methods, such as phenomenology, grounded theory, and interpretive description (e.g., Aldiabat, 2011; Maggs-Rapport, 2000; Thorne et al., 2004; Timmermans & Tavory, 2007). Hammersley (2006) observes that the term ethnography “is used in different ways on different occasions to mark off work of one kind from that of another” (p. 3) and suggests scholars should always clarify their usage of the term. Recognising this, we advocate for a plural research landscape in which different approaches to, and understandings of, ethnography can co-exist. Like Hammersley, we see the need for scholars to more clearly articulate which kind of ethnography they are utilising and its underlying assumptions (see also Trundle & Phillips, 2023). It is thus important in this article that we too define what we mean by ethnography.

Ethnography, we argue, is a research methodology that shapes both methods of fieldwork and methods of writing and communicating. As Campbell and Lassiter (2014) explain, being an ethnographer involves a particular commitment to “begin with people” and to understanding them on their own terms and in their own words, a mode of research that centres “the human relationships around which ethnography ultimately revolves” (p. 4). This makes ethnography a “deeply personal and positioned” encounter rather than a positivist encounter in which social distance seeks to prevent researcher bias. Ethnographers conceive of this encounter with participants as a collaboration, as co-learning between researchers and those whose lives they come to know through research. Meanings are thus co-constructed and interpretive.

As Campbell and Lassiter (2014) argue, ethnography also “grapples with the idea of culture, however deeply compromised” (p. 8). While many ethnographers find the concept of culture problematic, and resist reductive or immutable understandings of culture (e.g., Abu-Lughod, 2008; Chaudhuri-Brill, 2016; Ingold, 1993; Wright, 1998), the concept remains salient within ethnography, either implicitly or explicitly, as a tool to understand the diversity of “social meanings and activities” (Brewer, 2000, p. 11) within and across human groups, as well as a frame to foreground localised perspectives (Brewer, 2000, p. 17).

Ethnography, as we elaborate below, is as much a method of analysis and a writing practice as it is a method of data collection. It involves an open-ended, iterative, and creative mode of analysing and a richly descriptive and socially contextualised mode of writing that makes visible the personal and positioned nature of the research encounter (Coffey, 1999). The “steps” of ethnographic analysis and the “rules” for ethnographic writing are thus not easily codified or standardised (see Ballestero & Winthereik, 2021; McGranahan, 2020).

Our definition of ethnography purposefully does not centre immersive research in social settings and extended observation of social relations, as is commonly centred within many methodological definitions of ethnography (as we illustrate below). Our definition thus invites interview-based health ethnographers away from the periphery of ethnographic method and firmly into its centre.

## Our Contribution

In this article, we seek to extend and contribute to current understandings and practices of ethnographic interviewing in two ways. First, as noted above, well-cited texts on the ethnographic interview define it as necessarily intertwined with participant observation research. For example, Spradley (1979) defines the ethnographic interview as akin to an informal “friendly conversation”: “Skilled ethnographers often gather most of their data through participant observation and many casual, friendly conversations. They may interview people without their awareness, merely carrying on a friendly conversation while introducing a few ethnographic questions” (p. 56). Skinner (2012) also “recommend[s] the interview as a part of participant observation and not apart from participant observation” (p. 35). Correspondingly, Hammersley and Atkinson (2007) frame the interview within “participant oral accounts” and as supplementary to naturally occurring, unsolicited talk and participant observation. They position the interview as a tool to deepen the primary data of participant observation: “however skilful a researcher is in negotiating a role that allows observation of events, some information will not be available at first hand” (p. 98). But they caution: “there is an increasing tendency for qualitative research, even that labelled ethnographic, to rely exclusively on interviews … there are serious questions to be raised about this trend … Any tendency to assume that interviews are the default method of the ethnographer must be challenged” (Hammersley & Atkinson, 2007, p. 103).

We disagree that interviews alone are not sufficiently ethnographic. In this article, we outline how an ethnographic approach may be realised and applied within an interview study to produce robust insights. The “traditional” approach to the ethnographic interview proposed in the above-mentioned texts is out of step with contemporary research. Scholars within medical anthropology and medical sociology, as well as in health research more broadly, are increasingly performing ethnographic interviews outside of participant observation fieldwork. In a recent review of ethnographic research articles in medical anthropology journals and health science journals over the last decade, Trundle and Phillips (2023) found on average 44% of the research projects examined did not use participant observation.

This reflects, in part, the realities of research in health settings, where participant observation is not always possible nor always considered ethical (Rashid et al., 2015). It furthermore reveals the realities of contemporary health research funding and timeframes (Cruz & Higginbottom, 2013). It also reflects recent critiques that the ideal of long-term, immersive, and uninterrupted fieldwork is sometimes elitist, and often exclusionary of women, minorities, and those with care commitments (Günel et al., 2020). The rise of the ethnographic interview as a primary method indicates a clear need for a cogent description of the ethnographic interview in its own right, a method which may or may not be coupled with participant observation, making clear the distinctive dispositions of “being ethnographic” when approaching an interview (Madden, 2022).

Second, our approach to the ethnographic interview is distinct in not offering a detailed or programmatic guide to the types of questions ethnographers should ask. For example, Spradley (1979) offers a comprehensive 12-step guide to the ethnographic interview and categorises different types of questions, from grand tour to mini tour questions, and structural, descriptive, and contrast questions. Such an approach is endorsed by Madden (2022) in his influential book *Being Ethnographic*.

Rather than a programmatic, how-to guide to ethnographic interviews, we advocate for and offer an epistemic and methodological understanding of the ethnographic disposition that underpins the ethnographic interview. A strong understanding of these principles can then be applied to flexibly generate diverse ethnographic interview designs and techniques suited to the needs of specific projects and contexts.

## What Makes an Interview Ethnographic?

The following seven principles and accompanying examples articulate, we argue, the core features of an ethnographic disposition towards the interview.

### The Humility of Taking People’s Self-Narratives Seriously

Humility involves the researcher beginning from the presupposition that “There is a lot I don’t know” or “I am seeking to understand far more than I currently do.” This idea is sometimes equated with an outdated anthropological ideal of the researcher as “stranger,” an outsider position that allows the researcher to ask naïve questions that someone familiar with a community might overlook (e.g., Nash, 1963). We are not advocating this idea but rather a type of researcher humility in which, no matter how familiar a researcher feels they are with the community or subject of research, they remain committed to an open, explorative approach in which unexpected results are normalised and welcomed (Willis & Allen, 2011).

This is particularly important for health scholars with deep familiarity and expertise of, and even insider status within, their field sites. Even when the research question is focused, as it often is in health research, ethnographic interviews do not seek to confirm a hypothesis (Tammivaara & Enright, 1986). This is because a hypothesis not only determines the questions being asked but also narrowly delineates the answers possible.

This openness is linked to humility because it involves ceding control to the interviewee in shaping the direction of the ethnographic interview. Such an interview is characterised by the idea “that the researcher must first learn from their informants what the right questions to pose even are” (Maeder, 1995, p. 66) and that “both questions and answers must be discovered from the people being interviewed” (Westby, 1990; see also Bernard, 1988; Walford, 2007). Indeed, as Fetterman (1980) argues:

many books about interviewing … emphasize control. In formal and semi-structured interviews, maintaining control of the direction of the interview is useful to ensure that the interview produces the target information in the short time allotted. However, the ethnographer wants the interviewee to be in control much of the time. [Interviewers] taking charge of most interviews and maintaining control of them sacrifices too many data. (p. 57)

As Gilley (2010) explains, when an interviewee does not follow an expected social script, for example, interrupting and ignoring the interviewer, this is not necessarily a sign the interview is “going badly” or failing to gather data but potentially a sign that the interviewee is subverting the interview into their own site of communication and meaning. Acts of ignoring and interrupting their interviewer’s questions offer valuable and productive insights in their own right about what matters and what is at stake for the interviewee.

**Reflection – Humility**

*John Gardner*: I interviewed a very senior health professional at a major hospital organisation as part of a project exploring the impact of a new technology. As soon as the interview began, the participant spoke about a major initiative he had successfully spear-headed within his organisation, and continued to do so for 50 min with very little prompting or guidance from me. Afterward I considered dismissing much of what was said as being a not-directly-relevant self-promotional spiel. However I realised that the participant’s account could also be viewed as a forceful and passionate reflection of a worldview that, because of his senior position, would continue to have a significant impact on his organisation. It provided me with insights into the values and aspirations that were driving changes to healthcare – insights that only became apparent because the participant did not engage directly with my initial questions.

### Being Prepared to Revise Pre-Conceived Ideas of What the Problem Is or What a Phenomenon Is

As nursing scholar and ethnographer Muecke (1994) argues, health scientists are socialised to “think in terms of problem identification as a first step of inquiry” (p. 203). In her discussion of health sciences ethnographies and focused ethnographic methods, Muecke (1994) argues that it is crucial for the scholar utilising ethnographic methods to ask, “Does the preselection of the problem or phenomenon of study compromise the inductive nature of the study too much?” (p. 201). As noted in the first point, ethnographic rigour depends on “the openness of the field-worker to contrary interpretations … the greatest risk of focused ethnographies is that the boundaries of their focus unknowingly exclude what is relevant” (Muecke, 1994, p. 203; see also Bauman & Adair, 1992, p. 15). Muecke (1994) offers an example:

A student who wants to conduct a focused ethnography to find out why a particular ethnic group of women don’t breastfeed their children, for example, should be counselled either to change the question (e.g. broaden it to how do infants get nourished in that group and why they get nourished that way), or to change the methodology to one more appropriate to the research question. (p. 203)

Within focused questions, the practices under study can appear settled and remain conceptually unelaborated. For example, a framework of care might be applied to guide questions that predefine what “care” means. While this ontological common ground is often essential to medicine (White & Willis, 2002), it restricts the insights of an ethnographic approach. Ethnography means acknowledging cultural and social diversity, that different actors might understand, practice, or live core ideas, such as care, differently, and indeed, opening up such basic categories of action to reveal diversity and disjuncture can be crucial for understanding and addressing why stubborn health problems persist (Candea, 2019).

As Bauman and Adair (1992) argue, it is vital that “ethnographic interviewers avoid introducing their own words in an ethnographic interview and, whenever possible, repeat the informants’ own expressions when probing for more detail or additional examples” (p. 13). Rather than approaching language as a vehicle to access meaning, facts, and information about a topic, the creative use of language is meaningful in its own right. As Bauman and Adair (1992) argue, “treat the informant’s language as data. Language is a window to the ways individuals communicate cultural meanings; the words people use provide the structure and categorization of their experience” (p. 13).

At a more basic level, ethnographic interviewers should be prepared for and attentive of interviewees who have very different ideas about what an interview is, what it entails, and whether it is an appropriate means of interaction (Koven, 2014, p. 506). While the goals of interviewers are usually to gather knowledge to answer a research question and/or address a social problem, the motivation of the interviewee might be entirely different (Briggs, 1986, p. 39). For an ethnographer, this is not simply a problem to be circumvented through more sophisticated methodological design or by building better rapport. Rather, it is reflective of participants’ experiences and beliefs and is worthy of attention, understanding, and explication vis-à-vis the evolving research questions and direction. Thus, an ethnographic approach to interviews means acknowledging that when interviewees focus upon issues and experiences that at first seem extraneous to the research topic, their relevance often becomes apparent over time (Koven, 2014). The links that interviewees draw between the questions an interviewer asks, and their own lived experiences and concerns that they wish to share, should be taken seriously, even if the interviewer does not yet understand the logic and pertinence of those links.

**Reflection – Revising Pre-Conceived Ideas**

*Tarryn Phillips:* I needed to revise pre-conceived ideas when I conducted a research project with some colleagues – Celia McMichael and Michael O’Keefe – on a small island in Fiji. We were collaborating with a local doctor to better understand how Indigenous and Indo-Fijians made sense of the rapid and relatively recent rise of non-communicable diseases (NCDs) such as diabetes and high blood pressure in their communities. I arrived with my own preconceptions, which were that the NCD crisis was ultimately caused by broader forces beyond the community’s control, including the impact of colonisation on agriculture, diets and lifestyles, and the neoliberal food systems that disproportionately sold and marketed unhealthy products to them. I assumed people would agree with me, and that we would share outrage about this injustice. Yet, while all of the interviewees agreed that a change of diet was pivotal, they did not blame inequality for this shift. They tended to blame themselves, and each other. Voreqe felt that because women were now getting jobs at the local fishing cannery, they weren’t providing balanced diets for their family. Seru said that unemployed men were letting their families down because they hadn’t learnt how to cook. Vikash, an Indo-Fijian taxi driver, said that Indigenous people were more careless with their health than their Indo-Fijian brothers, and therefore were to blame for their own suffering (his Indigenous friend Aseri agreed with him). The overwhelming narrative was one of individual responsibility. As Una explained, “We invited the disease to come to us.” These narratives revealed a different and more subtle kind of injustice to the one I began looking for: the ways in which global public health discourses about individual responsibility infuse with gendered expectations and racialized stereotypes to take on particular local meaning.

As illustrated in the above reflection box, interviews allow the researcher to put aside their own preconceptions and take seriously people’s desires to narrate their lives in ways that position them as active agents. Narratives within health studies that cast them as passive victims of powerful forces do not always resonate with participants’ own sense of trying to exert control, develop strategies, and have influence over the forces shaping their lives.

### Attentiveness to Context

Discursive practices and speech acts are expressed and performed in particular environments. Ethnographic interviews require careful attention to the surrounding setting within which the interview takes place. Individual linguistic stories are rendered meaningful within social, physical contexts that foster and occlude particular speech patterns, actions, or identities (Ghodsee, 2016). Environments convey meaning, whether it be the peeling walls of a clinic, the broken lightbulb in a hall unchanged for weeks, the three-ply toilet paper in a marble bathroom, the close arrangement of vinyl chairs in a waiting room, or the curtains that remain open or shut on a ward.

Ethnographic interviews are often conducted in the everyday physical spaces of the interviewee’s world, for example, in their home, workplace, neighbourhood, the clinics they visit, or the community spaces in which they normally participate (Barker, 2012, p. 55; Burawoy et al., 1991). This allows interviewees familiarity and some control and meets them within the spatial and temporal flow of their ordinary lives. Such an approach allows us to observe firsthand the bodily modes of engagement and expertise of people vis-à-vis their environments (Müller, 2021, p. 48).

Ethnographic interviews are not only carried out in static modes but might also occur while walking, moving, and doing. Interviews in which action accompanies description attend to the material world and the body in situ (De Leon & Cohen, 2005). Talking while moving and doing allows interviewees to demonstrate their expertise and communicate beyond linguistic or memory-based registers. “Conversations situated within the lifeworld under investigation enable direct reference to the things on hand” (Müller, 2021, p. 48). In this sense, ethnographic interviews can be thought of as contextualised conversation (Stage & Mattson, 2003).

Placing speech within its lived context should not be reduced to a process of triangulation in which what is “said” is tested against what is “done” or observed to be done, in order to increase the accuracy of data (Kleine, 1990). This perspective assumes that there is a singular reality to be located. Situated interviews offer us something more nuanced than this because human narratives are never entirely coherent, nor do they simply record, more or less accurately, the world “out there.” Personal narratives are attempts to render the self and wider world meaningful, to exert agency over it, and even to create records of dissent against unwanted conditions or stories others tell about the self (Coffey, 1999). Thus, when words and actions don’t appear to “match up,” we are offered an opportunity to explore why and how this comes to be for individuals and to examine what makes certain actions doable but not sayable or sayable and not doable (Rinaldo & Guhin, 2022).

**Reflection – On Materiality in Interviews**

*John Gardner*: What do participants do with their mobile phones during an in-person interview? Most participants in research I’ve undertaken have kept their phones hidden, sometimes placing it in a bag or pocket after having carefully turned it to silent mode. Some participants – particularly clinical consultants – have put their phone on the desk or table in front of them, noticeably keeping an eye on it for alerts. This creates an interesting dynamic in the interview, especially when participants “need to take this call.” For me, witnessing this has provided some insight into the structure and flow of day-to-day collaborative clinical work. I’ve seen the dynamic play-out in multidisciplinary clinical meetings, the rhythm of which is partly guided by consultants needing to answer their phones, while other health and allied professionals patiently wait for their return.

**Reflection – On Materiality in Interviews**

*Catherine Trundle*: When I arrived at Tessa’s house to interview her, she was well-prepared. She must have taken some time before my arrival to drag out the heavy white boxes of documents and arrange them on the floor of her lounge. Without thinking about it, I sat down on the carpet next to her, and this was where we conducted the interview.

“This is it,” she told me. “All of it.” She didn’t need to say much else. For 10 min, she let me leaf through the mountains of documents, all from government veteran agencies. She was in no hurry to start the formal interview. I could tell she wanted me to experience the weight of these documents with my own body. To observe their official and impersonal tone, the never-ending requests for further evidence or assessments.

When we did turn on the voice recorder and begin to talk, she kept gesturing to the documents around us, as proof. Of how the government had failed to look after her late husband as he sought pension support for radiation related illnesses that he attributed to his military service. Through the materiality of paper, she wanted me to understand the slow bureaucratic violence of their struggles.

The rise of online communication, social media, and virtual social spaces also necessitates new practices of interviewer attentiveness that challenges many traditional ethnography ideas of the contextualised conversation. Virtual worlds and online interaction have become meaningful and powerful dimensions of people’s lives and social worlds (Barker, 2012; Hallett & Barber, 2014). Ethnographic interviewing has been adapted to include different digital forms of asking and answering questions that engage participants synchronously and asynchronously (e.g., Skågeby, 2011; Snodgrass, 2016). Rather than seeing digital interviews as lacking contextual detail, an ethnographer can consider the unique specificities of digital and virtual contexts and the ways in which they enable and shape the social interaction of an ethnographic interview (Boellstorff, 2015).

As Crichton and Kinash (2003) argue, in asynchronous online text-based ethnographic interviews, an interviewee “has the time and access to information resources, to inform, reflect, revise, and iterate responses” (p. 9). They can send links, pictures, or quotes that help elaborate their thoughts and contextualise them within larger flows of information and in relation to wider experiences and communities. In synchronous video interviews, participants can screenshare or add links or information to the chat while talking. Walking interviews can occur during a video interview or in a virtual world. In walking video interviews, where the interviewee chooses to point the camera, what objects they include or exclude from the screen, and what happens on or off camera provide rich contextual information complementing verbal utterances (Hallett & Barber, 2014). As Howlett (2022) notes, ethnographic interviews via video can in fact offer glimpses into different spaces and times in a participant’s life not afforded face-to-face. In shifting from face-to-face to Zoom interviews during the COVID-19 pandemic, Howlett noticed her participants were more likely and more comfortable to show their private home spaces, in part because they were more in control of what and how much they shared on video. Howlett also saw participants’ lives at more varied hours of the day. Critical reflexivity is crucial in understanding the insights about context that different kinds of ethnographic interviews offer and enable (Zhao & Li, 2023). As the following reflection illustrates, videoconference interviews can provide insights into the ways in which our participants’ everyday world are mediated and shaped through digital technology.

**Reflection – Interviews via Video-Conferencing**

*John Gardner*: One junior clinician with an administrative role was video-conferencing the interview in a busy open-plan office. He was wearing headphones and appeared to be hunched over his screen, and there was a great deal of background noise and movement. It was clearly a busy, loud environment. He said that much of his administrative role involved video conference meetings very similar to this one – he had to find space in a hectic office setting, bring his headphones to try and block out the noise, and use the blur function to reduce visual distractions behind him. This contrasted with the sense of seniority and power conveyed by the video conferencing arrangement of other clinicians I interviewed. These clinicians worked in the same building as the junior clinician, but their offices were quiet (no headphones were necessary), backgrounds were seldom blurred, and in some cases, it was possible to discern that their spaces had been personalised with framed photos or pictures. These clinicians, I envisaged, commanded much more authority when video conferencing with colleagues.

### The Undeniable Relationality of Interviews

Ethnographic interviewing requires the interviewer to acknowledge that they are not, and never can be, a neutral presence in the interview (Lumsden, 2014). The ethnographic interview is, at its core, a social relationship, albeit sometimes a fleeting one. As a social relationship, it contains a reciprocal dynamic and is mutually performative; participants often desire responses from us that expose our views or relationship to the topic discussed, test our trustworthiness, or test our legitimacy to ask the questions we ask (Briggs, 1986). Indeed, interviewees often attempt to elicit subtle responses from the interviewer and, at the very least, they watch us carefully to gauge our reactions to their perspectives and experiences and recalibrate their responses accordingly. “Interviewers must give feedback during the interview, confirming that the message has gotten through” (Westby, 1990, p. 105; see also Hammersley & Atkinson, 2007), that is, that the interviewee has been understood on their own terms.

In talking with participants about their worlds, we unavoidably enter as actors into the social worlds they describe. We are thus not external witnesses to the issues interviewees discuss. By participating in their reflections of their worlds, we have an effect on that world. As Ewing (2006) argues, “Speech production is an act that creates, sustains, and disrupts relationships. It changes the world and one’s position in it. While conducting research, the ethnographer is inevitably drawn up into this process of negotiation” (p. 91). Acknowledging this means framing ethnographic interviews as “talking partnerships” (Rapport, 2012) or as a process of “coinvestigation” (DeVault & McCoy, 2001). Put another way, in contrast to approaching interviewing as a process of “being there,” ethnographic interviews necessitate a “being with” interlocutors (Hockey & Forsey, 2012, p. 75).

For the ethnographic interviewer, the positivist goal of erasing the “effect” of the interviewer on the researched, or seeing the interviewer’s presence as a source of data “interference” to be countered, is naïve (Lumsden, 2014). Instead, Haraway’s (1988) understanding of the inherently “situated” nature of knowledge production underpins an ethnographic approach. Reflexivity is thus crucial to the ethnographic interviewer, not as a tool to test “validity,” to banish “bias,” or to neutralise the effects of the researcher but as an ongoing process of bringing to light the positionality of both the interviewer and interviewee, to understand what such positionalities enabled and foreclosed in the co-production of interview narratives (Fina, 2019; Marques da Silva & Webster, 2018).

Ethnographers do not see this inherent relationality as a feature specific to ethnographic interviews but as an undeniable feature of all interviews and indeed research more generally (Lumsden, 2014). What is essential to an ethnographic interview, however, is a comprehensive commitment to disclosure of this relational reality (Coffey, 1999). This disclosure goes beyond a few sentences that state the possible effects of the interviewer’s and interviewee’s gender, class, age, sexuality, or ethnicity might have had on the interview data and how this was “countered.” It involves, first, a desire to complexly understand when and how the different positionalities of the interviewer and interviewee mattered, intersected, and shifted to shape the conversation at different points (Coffey, 1999). Second, it involves a recursive commitment from the interviewer to examine their intellectual and ontological commitments as they are trying to make sense of the data (Wacquant & Bourdieu, 1992). And third, it requires they attempt to frankly write their presence within the interview into their academic writing, thus avoiding slipping into the invisible, authoritative narrator voice (Tedlock, 1991).

**Reflection – Relationality, and Embracing Complexity and Contradiction**

*Catherine Trundle*: During research on the health claims of British veterans who had participated in nuclear experiments, I found veterans continued to test me. Was I sympathetic to their cause or was I going to publish findings that might harm their claims against the government? Over a decade of research, I needed to prove my trustworthiness on multiple occasions. Gaining participants’ insights and understanding their perspectives meant eschewing a neutral and disinterested researcher stance.

When observing the test veterans’ case against the Ministry of Defence in the High Court of London, where I sat mattered. The first day I entered the court, a widow, Teresa, beckoned me over. She was sitting behind the defence’s QC, along with several other veterans. She liked to sit there, to remind the government who they were dealing with. Sitting next to her, I was able to observe at close range the way in which she held her body upright, face grave, staring defiantly at the defence.

During the lunch break, the widows invited me to sit with them in the High Court cafeteria. I had also been interviewing lawyers for the research, but it was clear that the widows expected me to sit with them. We watched the lawyers shuffle into a private room for a catered lunch. The widows rolled their eyes, noting the cost to the taxpayers of the Ministry of Defence’s legal defence. Along with a sympathetic journalist, I bought the ladies sandwiches. They survived on small pensions, and the train journey to court from their respective homes outside of London was never cheap. Teresa patted my hand, then began to talk about the effects of the case on her, the other widows she knew, their anger and frustration, the mixture of hope and cynicism, their strategies for supporting each other and the sense of community that had formed amongst them during the case.

“I worry about some of the veterans, after the court-case is finished, the loneliness,” a widow, Grace reflected. In these conversations, I was able to observe the complex, contradictory effects of the legal case, simultaneously detrimental and supportive of their wellbeing, a topic I followed up in formal interviews.

### Embracing Complexity and Contradiction

An ethnographic disposition is not aiming to reduce the messiness of lives into coherent and seamless data themes. Instead, its commitments are towards respecting “the irreducibility of human experience” (O’Reilly, 2009, p. 3). In this way, ethnography recognises that people’s stories of their lives are often woven through with contradictions. As Ewing (2006) argues, the interviewee “is often ambivalent and caught in conflicting positioning that leaves traces in the interview process that attempts to both reveal and conceal at each point in the conversation” (p. 93). Ethnographic interviewers do not try to smooth or resolve such complexity and contradictions through questioning, to work out the “real,” or to hone in on the coherent view. Rather, they take such complexity seriously and allow it to persist within the conversation (Ewing, 2006).

One way in which complexity is encouraged is through making space for vivid description of lifeworlds, for the collection of sensorially rich descriptions (Pink, 2015). This often entails slowing the flow of the ethnographic interview down in order that reflections or descriptions of events can be dwelled within for longer. Probing questions encourage the interviewee to return to a point just made, to articulate and inhabit it further, from different experiential standpoints, rather than explain “what happened next,” or move swiftly to explaining why it happened (Westby et al., 2003).

A commitment to complexity is also achieved through allowing or encouraging the interviewee to move between points of view. The ethnographic notions of emic and etic is commonly reduced to a simplistic dichotomy, between “insider” participant/lived perspective and outsider researcher/academic perspective (Levy & Hollan, 2015). By contrast, the ethnographic interview offers an opportunity for the interviewee to tack back and forth between a discussion of their unique individual experience of a phenomenon, and their views of how the phenomenon occurs more generally, the difference between *participant* and *observer* positions, or between subjective point of view and the role of “expert witness” to the world around them (Levy & Hollan, 2015). Attending to these two narrative positions, and the spaces between them, “illuminates the … conflicts, coherences, and transformations … between the woman-in-herself (either in her own conception, or in the interviewer’s emerging one) and aspects of her perception and understanding of her external context or life-world” (Levy & Hollan, 2015, p. 5).

The ethnographic interview narrative is not, however, a simple movement between subjective and objective view, inside and outside, and individual versus culture perspective (Coffey, 1999). Both participant and observer positions require the interviewee to turn personal experiences and the external world into narrative objects in diverse ways, and both require an interviewee to inhabit different types of subject positions, such as the different roles they occupy in different social contexts or moments of life. In other words, rather than try to know the person through the narrow lens of a research population group (as, for example, a patient, a doctor, or a parent), ethnographic interviews allow the interviewee to reflect upon the multiple roles they occupy in everyday life (Beals et al., 2020; Westby et al., 2003).

Indeed, as ethnographers (Jacobs-Huey, 2002; Narayan, 1993) have made clear, the notion of insider versus outsider viewpoints is simplistic, and insider/outsider status often involves gradations, contestation, and shifts. For this reason, many ethnographers have rejected the emic/etic idea and instead advocate for subtle descriptions of the multiple, dynamic, and shifting positionality of both the researcher and the interviewee (Song & Parker, 1995).

A further way in which ethnographers embrace complexity is by conducting follow-up interviews and conversations. Thus, seeking further elaboration, staying in touch, and having clarification conversations are commonplace. Stage and Mattson (2003) show that ethnographic interviews “require researchers to pause and reflect on context and include research participants in a reciprocal process” of meaning-making (p. 103). As DeVault and McCoy (2001) argue, ethnographic interviews not only produce answers to research questions but also produce new lines of inquiry about ongoing action and reshaping and generating new questions:

The term ethnography highlights the importance of research methods that can discover and explore … everyday activities and their positioning within extended sequences of action … The interviewer’s goal is to elicit talk that will not only illuminate a particular circumstance but also point toward next steps in an ongoing, cumulative inquiry. (p. 751)

An attention to complexity lies at the heart of how ethnographic interviews transform into ethnographic writing, our next point.

### The Ethnographic Interview in Ethnography Writing

Approaching interviews ethnographically extends beyond the data gathering stage of research and includes transforming interviews into ethnographic texts. Ethnographers seek to bring interview data to life for the reader through ethnographic writing, spotlighting specific lives and individual case studies within their wider context (Ghodsee, 2016). Ethnographers conceptualise interviews as a window into people’s specific, complex, and embodied experiences of a phenomenon rather than solely the collection of material to illustrate broader patterns in particular populations. As McGranahan (2020) explains, “When we read an ethnography, we expect to meet people, not just categories of them” (p. 6).

When transforming interviews into ethnographic texts, ethnographers often use literary conventions to render insights more visible or powerful. Ethnographers often begin an article with a descriptive vignette, which introduces an interviewee in a way that is a vivid and emotionally resonant entrée into the topic (Trundle & Phillips, 2023). Such rich descriptions of interviewees’ lives often extend beyond the details that are *directly* relevant (such as the symptoms of illness or particular interactions with doctors) to include wider aspects of their daily lives that reveal a unique human being in all their complexity (Loblay et al., 2021). Consider, for example, the first paragraph of this article, in which Catherine Trundle is writing about the aforementioned quest of British veterans to receive compensation for workplace exposure to toxic radiation. Rather than beginning with statistics or broader context, or details about the veterans’ health condition, Trundle offers an intimate description of the interview with Tessa (described earlier in the fourth reflection box):

Piles of documents. Stack upon stack of official letters signifying an almost endless back and forth exchange with state agencies. Tessa had dragged them out of their boxes to impress upon me the weighty, officious nature of bureaucratic care and its daily grind. I sat with her on the floor of her lounge, surrounded by these small paper towers….

Here, Trundle invites readers to sit with her in the sensorial aspects of the interview. This has the effect of providing insights into the participant’s lived experiences that exceed the words uttered during the interview.

Another ethnographic technique for writing up interviews is juxtaposition (Hall, 2010). This involves placing contrasting comments or behaviours from participants alongside each other in a way that renders the contradictions between them starker. The capacity for such contrast is embedded at the outset of many ethnographic projects, which are often designed to capture voices from multiple perspectives on an issue – comparing the views of doctors and patients about what care should look like, for example, or revealing differences in how bureaucrats and people at the front-line approach decision-making about a vulnerable population.

**Reflection – On the Use of Juxtaposition**

*Tarryn Phillips:* I find that juxtaposing excerpts from interviews is iterative for both analysis and writing. As I begin to collate material from a research project, contrasting interviews in relation to one another can be a useful way to reveal what is most meaningful to participants and which problems seem to be the sticking points. For example, I utilised juxtaposition in one article about how experts diagnose medically uncertain conditions. The article begins with quotes from two experts who arrived at fundamentally different diagnoses for a group of workers who alleged to have developed chemical injuries at a mine site. The first was a clinical psychologist who felt sympathetic to a chemical explanation:

I’ve interviewed many, many [chemically sensitive people] […] I’ve seen them first-hand. I’ve visited them in their homes, and know how much they suffer. So that’s why I’m prepared to take a stand. (Phillips, 2010, p. 1026)

This is followed by the views of a sceptical infectious disease specialist, who questions the workers’ motivations and medical legitimacy:

I think these [workers] get a whiff of compensation rather than a whiff of chemicals [. . .] They have an illness of some sort which cannot be diagnosed. They look around, they say, “What is the cause of this? My God, what’s that smell? That must be the cause.” And then they switch on Foxtel, what do you know? Erin Brockovich is on TV. (Infectious disease specialist) (Phillips, 2010, p. 1026)

The contradiction between these views immediately and dramatically reveals the heart of the issue, which is that doctors approach medical uncertainty differently, depending on their disciplinary standpoint, individual evidentiary standards, and moral orientation towards their professional role.

As these examples illustrate, using interviews ethnographically involves weaving insights and the presence of participants throughout a text, rather than confining them to the findings section (Loblay et al., 2021). Importantly, this is dependent on journal writing conventions, as some disciplines allow more flexibility in how people structure the narrative than others.

### Knowing the History of the Method and Considering the Power Dynamics Involved

It is easy to conceptualise a method outside of its historical or political context, as a set of neutral and technical procedures onto which political and social concerns have been overlaid at different points in time and in different settings. Instead, we urge scholars to consider the “baked in” history of ethnography and interviewing that continues to shape how particular communities react to researchers with mistrust, trust, scepticism, or hope (Smith, 1999). It is crucial to consider how the power dynamics of an ethnographic interview, so often a “speech act external to community social relations” (Gilley, 2010, p. 113) play out in a particular community. This is particularly vital in medical and health spheres where unequal hierarchies of authority, social influence, vulnerability, and agency are variously present. As Koven (2014) argues, “scholarly attention to interviews should go beyond methodological questions about how to conduct and analyse interviews ‘better’ to also address epistemological issues about the communicative hegemony of interviewing among those who live in ‘interview societies,’ where interviewing and interview-like practices are ubiquitous” (p. 500) (see also Briggs, 1986).

Interviews do not produce neutral truths but rather “authoritative and consequential knowledge about groups and individuals” (Briggs, 2001; Koven, 2014, p. 499). The use of ethnographic knowledge within colonialism to support imperial rule and indigenous dispossession is a stark case in point (Asad, 1973; Smith, 1999), as was the use of research interviews to pathologise and stigmatise gay men in the 1940s, 1950s, and 1960s (Kong et al., 2001). “Research fatigue” amongst communities who are repeated targets of interview research is also well-documented (e.g., Clark, 2008), summed up by one participant in a study by Way (2013): “I am interviewed over and over and over and nothing has ever changed” (p. 1).

Ongoing reflection regarding the power dynamics and potential for exploitation is also crucial (Townsend & Cushion, 2021). This allows the interviewer to actively consider how they will respond to the traditional asymmetrical power relations between the interviewer and the interviewee (for examples of such strategies, see Bartlett et al., 2007; Chacaby & Plummer, 2016; Mishler, 1986). As feminist ethnographer Stacey (1988) argued, however, scholars often assume that a detached, hierarchical research style is the most likely to create problematic power dynamics between researchers and those they research. But as she points out regarding feminist methodological debates, close bonds, non-hierarchical relational modes, a commitment to ongoing engagements and trusting ties between the interviewer and the interviewee do not, in themselves, counter exploitation. As she argues, seemingly equitable and trusting relations between ethnographers and participants can exploit participants by masking the role and intentions of the researcher and the ultimately extractive dynamics of much research that gathers life experiences as “data” and utilises it for academic knowledge production outside of the communities from which it originates.

**Reflection – The Politics and History of Interviews**

*Catherine Trundle:* Between 2006 and 2007, I conducted doctoral research in Florence, Italy, on charitable services for migrant groups. By first embedding my research amongst charity workers, the recipients of charity came to identify me strongly with the role of charity giver and with Christian charity services. When I sought to interview charity recipients, I was met by stony silences or gentle excuses. Many were happy to talk with me informally, but interviews proved difficult, and I soon abandoned interviews as a method.

I came to understand that, as many participants were undocumented, they associated interviews with degrading social services appointments in which they were compelled to provide evidence of need to access entitlements. Interviews were also associated with interrogation by the police, and with arduous *denuncia* practices by which trafficked women could gain legal immigration status by providing testimonies to help prosecute their traffickers. Many viewed interview type events as a direct threat to their ability to live peacefully, “under the radar” from authorities.

## Conclusion

Ethnography has travelled outside of anthropology and sociology into wider interdisciplinary use, and the ethnographic interview is an increasingly common method in health research. Diverse scholars have adapted the ethnographic interview to fit with the needs and nuances of scholarship in each disciplinary setting, including health research settings (e.g., Cruz & Higginbottom, 2013). To support ethnographic interviewers in health to conduct robust research and to articulate the strengths of their method, we have offered an epistemological and methodological definition of the ethnographic interview that focuses on core ethnographic dispositions.

By grounding our discussion in the epistemic dimension of research, the seven dispositions argued for above provide an orientation towards interviewing that is responsive and open to innovation, without limiting or standardising ethnographic interviews. An ethnographic orientation – which includes approaching an interview with humility; a readiness to revise core assumptions about a research topic; attentiveness to context; relationality; openness to complexity; an attention to ethnographic writing; and a consideration of the politics and history of the method – allows researchers to achieve ethnographic depth while also conducting their interviews in a way that suits their unique research projects.

Being explicit about what makes an interview ethnographic helps to decentre participant observation and avoids casting the interview as ancillary or supporting data to the “real” observations. Such a hierarchy is unrealistic in the contemporary research landscape, and many ethnographers now rely mostly on interviews (Trundle & Phillips, 2023).

By articulating what makes an interview ethnographic, we can simultaneously offer concrete instruction to postgraduates embarking on their fieldwork and people new to ethnography, while also furnishing experienced ethnographers with justification to disciplinary gatekeepers about the ways in which interviewing is a valid and comprehensive ethnographic method in its own right. Increasingly, journal editors are requesting that qualitative health researchers prove the quality and rigour of their research through responding to appraisal checklists, such as the COREQ tool outlined by Tong et al. (2007), and qualitative research is systematically appraised using such tools as the Joanna Briggs Institute (2017) Checklist for qualitative research. Such checklists emphasise a reflexive and coherent methodological approach, and congruity between the epistemological and methodological approach with the chosen methods, analysis, and writing style. As Roulston (2019) argues:

By demonstrating a willingness to interrogate their epistemological positions in their approaches to interviews and outline what these imply for doing work of quality, researchers are better positioned to argue for the rigor, credibility, and validity of research findings. (p. 18)

Responding to these imperatives, the seven elements of the ethnographic disposition we have laid out here develop a language for ethnographers to justify the rigour of their interviewing methodology and method, and articulate its contribution to knowledge for interdisciplinary audiences. In this way, we hope to support the visibility, legitimacy, and rigour of the ethnographic interview within health research.

## References

[bibr1-10497323241241225] Abu-Lughod L. (2008). Writing against culture. In T. Oakes & P. L. Price (Eds.), The cultural geography reader (pp. 62–71). Routledge.

[bibr2-10497323241241225] Aldiabat K. (2011). Clarification of the blurred boundaries between grounded theory and ethnography: Differences and similarities. Turkish Online Journal of Qualitative Inquiry, 2(3), 1–13. 10.17569/tojqi.04785

[bibr3-10497323241241225] Asad T. (1973). Introduction. In Asad T. (Ed.), Anthropology & the colonial encounter (pp. 9–19). Ithaca Press.

[bibr4-10497323241241225] Ballestero A. Winthereik B. R. (2021). Experimenting with ethnography: A companion to analysis. Duke University Press. 10.1215/9781478013211

[bibr5-10497323241241225] Barker J. (2012). The ethnographic interview in an age of globalization. In Fardon R. Harris O. Marchand T. H. J. Nuttall M. Shore C. Strang V. Wilson R. A. (Eds.), The Sage handbook of social anthropology (pp. 54–68). Sage. 10.4135/9781446201077

[bibr6-10497323241241225] Bartlett J. G. Iwasaki Y. Gottlieb B. Hall D. Mannell R. (2007). Framework for Aboriginal-guided decolonizing research involving Métis and First Nations persons with diabetes. Social Science & Medicine, 65(11), 2371–2382. 10.1016/j.socscimed.2007.06.011 17689163

[bibr7-10497323241241225] Bauman L. J. Adair E. G. (1992). The use of ethnographic interviewing to inform questionnaire construction. Health Education Quarterly, 19(1), 9–23. 10.1177/109019819201900102 1568876

[bibr8-10497323241241225] Beals F. Kidman J. Funaki H. (2020). Insider and outsider research: Negotiating self at the edge of the emic/etic divide. Qualitative Inquiry, 26(6), 593–601. 10.1177/1077800419843950

[bibr9-10497323241241225] Bernard R. H. (1988). Research methods in cultural anthropology. Sage.

[bibr10-10497323241241225] Boellstorff T. (2015). Coming of age in second life: An anthropologist explores the virtually human. Princeton University Press. https://muse.jhu.edu/book/64529

[bibr11-10497323241241225] Brewer J. (2000). Ethnography. McGraw-Hill Education.

[bibr12-10497323241241225] Briggs C. L. (1986). Learning how to ask: A sociolinguistic appraisal of the role of the interview in social science research. Cambridge University Press. 10.1017/CBO9781139165990

[bibr13-10497323241241225] Briggs C. L. (2001). Interviewing, power/knowledge, and social inequality. In Gubrium J. Holstein J. (Eds.), Handbook of interview research (pp. 243–254). Sage. 10.4135/9781412973588

[bibr14-10497323241241225] Burawoy M. Burton A. Ferguson A. A. Fox K. J. Gamson J. Gartrell N. Hurst L. (1991). Ethnography unbound: Power and resistance in the modern metropolis. University of California Press.

[bibr15-10497323241241225] Campbell E. Lassiter L. E. (2014). Doing ethnography today: Theories, methods, exercises. John Wiley & Sons.

[bibr16-10497323241241225] Candea M. (2019). Comparison in anthropology. Cambridge University Press. 10.1017/9781108667609

[bibr17-10497323241241225] Chacaby M. N. Plummer M. L. (2016). A two-spirit journey: The autobiography of a lesbian Ojibwa-Cree elder. University of Manitoba Press.

[bibr18-10497323241241225] Chaudhuri-Brill S. (2016). Úloha antropologie v rozvíjení “konceptu kultury” ve veřejném diskurzu [The role of anthropology in developing the “culture concept” in public discourse]. Český Lid, 103(3), 323–370. 10.21104/CL.2016.3.02

[bibr19-10497323241241225] Clark T. (2008). ‘We’re over-researched here!’: Exploring accounts of research fatigue within qualitative research engagements. Sociology, 42(5), 953–970. 10.1177/0038038508094573

[bibr20-10497323241241225] Coffey A. (1999). The ethnographic self: Fieldwork and the representation of identity. Sage Publications. 10.4135/9780857020048

[bibr21-10497323241241225] Cook K. E. (2005). Using critical ethnography to explore issues in health promotion. Qualitative Health Research, 15(1), 129–138. 10.1177/1049732304267751 15574720

[bibr22-10497323241241225] Crichton S. Kinash S. (2003). Virtual ethnography: Interactive interviewing online as method. Canadian Journal of Learning and Technology, 29(2), 1–12. 10.21432/t2w02t

[bibr23-10497323241241225] Cruz E. V. Higginbottom G. (2013). The use of focused ethnography in nursing research. Nurse Researcher, 20(4), 36–43. 10.7748/nr2013.03.20.4.36.e305 23520711

[bibr24-10497323241241225] De Leon J. P. Cohen J. H. (2005). Object and walking probes in ethnographic interviewing. Field Methods, 17(2), 200–204. 10.1177/1525822X05274733

[bibr25-10497323241241225] DeVault M. McCoy L. (2001). Institutional ethnography: Using interviews to investigate ruling relations. In Gubrium J. F. Holstein J. A. (Eds.), Handbook of interview research (pp. 751–776). Sage.

[bibr26-10497323241241225] Ewing K. P. (2006). Revealing and concealing: Interpersonal dynamics and the negotiation of identity in the interview. Ethos, 34(1), 89–122. 10.1525/eth.2006.34.1.089

[bibr27-10497323241241225] Fetterman D. M. (1980). Ethnography: Step-by-step. Sage.

[bibr28-10497323241241225] Fina A. (2019). The ethnographic interview. In Tusting K. (Ed.), The Routledge handbook of linguistic ethnography (pp. 154–167). Routledge.

[bibr29-10497323241241225] Ghodsee K. (2016). From notes to narrative: Writing ethnographies that everyone can read. University of Chicago Press.

[bibr30-10497323241241225] Gilley B. J. (2010). “A balance of authority”: Ponca women’s cultural autonomy through the appropriation of the ethnographic interview. Intertexts, 14(2), 113–122. 10.1353/itx.2011.0006

[bibr31-10497323241241225] Günel G. Varma S. Watanabe C. (2020, June 9). A manifesto for patchwork ethnography. Member voices. Fieldsights. https://culanth.org/fieldsights/a-manifesto-for-patchwork-ethnography

[bibr32-10497323241241225] Hall S. (2010). Picturing difference: Juxtaposition, collage and layering of a multiethnic street. Anthropology Matters, 12(1), 1–17. 10.22582/am.v12i1.187

[bibr33-10497323241241225] Hallett R. E. Barber K. (2014). Ethnographic research in a cyber era. Journal of Contemporary Ethnography, 43(3), 306–330. 10.1177/0891241613497749

[bibr34-10497323241241225] Hammersley M. (2006). Ethnography: Problems and prospects. Ethnography and Education, 1(1), 3–14. 10.1080/17457820500512697

[bibr35-10497323241241225] Hammersley M. Atkinson P. A. (2007). Ethnography: Principles in practice. Routledge. 10.4324/9780203944769

[bibr36-10497323241241225] Haraway D. (1988). Situated knowledges: The science question in feminism and the privilege of partial perspective. Feminist Studies, 14(3), 575–599. 10.2307/3178066

[bibr37-10497323241241225] Hockey J. Forsey M. (2012). Ethnography is not participant observation: Reflections on the interview as participatory qualitative research. In Skinner J. (Ed.), The interview: An ethnographic approach (pp. 69–87). Berg.

[bibr38-10497323241241225] Howlett M. (2022). Looking at the ‘field’ through a zoom lens: Methodological reflections on conducting online research during a global pandemic. Qualitative Research, 22(3), 387–402. 10.1177/1468794120985691 35663097 PMC9095994

[bibr39-10497323241241225] Ingold T. (1993). The art of translation in a continuous world. In Palsson G. (Ed.), Beyond boundaries: Understanding, translation, and anthropological discourse (pp. 210–230). Berg.

[bibr40-10497323241241225] Jacobs-Huey L. (2002). The natives are gazing and talking back: Reviewing the problematics of positionality, voice, and accountability among “native” anthropologists. American Anthropologist, 104(3), 791–804. 10.1525/aa.2002.104.3.791

[bibr41-10497323241241225] Joanna Briggs Institute . (2017). The Joanna Briggs Institute critical appraisal tools for use in JBI systematic reviews. Checklist for qualitative research. https://joannabriggs.org/research/critical-appraisal-tools.html

[bibr42-10497323241241225] Kian M. Beach D. (2019). Implications of ethnography research method in educational and health studies. Social Behavior Research & Health, 3(2), 419–427. 10.18502/sbrh.v3i2.1788

[bibr43-10497323241241225] Kleine M. (1990). Beyond triangulation: Ethnography, writing, and rhetoric. Journal of Advanced Composition, 10(1), 117–125. https://www.jstor.org/stable/20865703

[bibr44-10497323241241225] Kong T. Mahoney D. Plummer K. (2001). Queering the interview. In F Gubrium J. Holstein J. A. (Eds.), Handbook of interview research. Sage. 10.4135/9781412973588

[bibr45-10497323241241225] Koven M. (2014). Interviewing: Practice, ideology, genre, and intertextuality. Annual Review of Anthropology, 43(1), 499–520. 10.1146/annurev-anthro-092412-155533

[bibr47-10497323241241225] Leder Mackley K. Pink S. (2013). From emplaced knowing to interdisciplinary knowledge: Sensory ethnography in energy research. The Senses and Society, 8(3), 335–353. 10.2752/174589313X13712175020596

[bibr48-10497323241241225] Levy R. I. Hollan D. W. (2015). Person-centered interviewing and observation. In Bernard H. R. Gravlee C. C. (Eds.), Handbook of methods in cultural anthropology (pp. 333–364). Rowman & Littlefield.

[bibr49-10497323241241225] Loblay V. Conte K. P. Grøn S. Green A. Innes-Hughes C. Milat A. Persson L. Williams M. Mitchell J. Hawe P. (2021). The weight of words: Co-analysis of thick ethnographic description and “friction” as methodological strategies in a health policy research partnership. Qualitative Health Research, 31(4), 754–766. 10.1177/1049732320962438 33034251

[bibr50-10497323241241225] Lumsden K. (2014). ‘You are what you research’: Bias and partisanship in an ethnography of boy racers. In K. Lumsden & A. Winter (Eds.), Reflexivity in criminological research: Experiences with the powerful and the powerless (pp. 275–286). Palgrave Macmillan. 10.1057/9781137379405_21

[bibr51-10497323241241225] Madden R. (2022). Being ethnographic: A guide to the theory and practice of ethnography. Sage. 10.4135/9781529716689

[bibr100-10497323241241225] Maeder C. (1995). In totaler Gesellschaft. Eine ethnografische Untersuchung zum offenen Strafvollzug *[In total society: An ethnographic investigation into the open prison system]*. Difo.

[bibr52-10497323241241225] Maggs-Rapport F. (2000). Combining methodological approaches in research: Ethnography and interpretive phenomenology. Journal of Advanced Nursing, 31(1), 219–225. 10.1046/j.1365-2648.2000.01243.x 10632812

[bibr53-10497323241241225] Marques da Silva S. Webster J. P. (2018). Positionality and standpoint. In Beach D. Bagley C. Marques da Silva S. (Eds.), The Wiley handbook of ethnography and education (pp. 501–512). John Wiley and Sons. 10.1002/9781118933732.ch22

[bibr54-10497323241241225] McGranahan C. (Ed.). (2020). Writing anthropology: Essays on craft and commitment. Duke University Press. 10.1215/9781478009160

[bibr55-10497323241241225] Mishler E. G. (1986). Research interviewing: Context and narrative. Harvard University Press. 10.2307/j.ctv26070x9

[bibr57-10497323241241225] Muecke M. A. (1994). On the evaluation of ethnographies. In Morse J. M. (Ed.), Critical issues in qualitative research methods (pp. 187–209). Sage.

[bibr58-10497323241241225] Müller F. (2021). Design ethnography: Epistemology and methodology. Springer Nature. 10.1007/978-3-030-60396-0

[bibr59-10497323241241225] Narayan K. (1993). How native is a “native” anthropologist? American Anthropologist, 95(3), 671–686. 10.1525/aa.1993.95.3.02a00070

[bibr60-10497323241241225] Nash D. (1963). The ethnologist as stranger: An essay in the sociology of knowledge. Southwestern Journal of Anthropology, 19(2), 149–167. 10.1086/soutjanth.19.2.3629165

[bibr61-10497323241241225] O'Reilly K. (2009). Key concepts in ethnography. Sage. 10.4135/9781446268308

[bibr349-10497323241241225] Phillips T. (2010). Debating the legitimacy of a contested environmental illness: A case study of multiple chemical sensitivities (MCS). Sociology of Health & Illness, 32(7), 1026–1040. 10.1111/j.1467-9566.2010.01255.x 21039616

[bibr63-10497323241241225] Pink S. (2015). The sensoriality of the interview: Rethinking personal encounters through the senses. In S. Pink (Ed.), Doing sensory ethnography (pp. 73–93). Sage. 10.4135/9781446249383

[bibr64-10497323241241225] Rapport N. (2012). The interview as a form of talking-partnership: Dialectical, focussed, ambiguous, special. In Skinner J. (Ed.), The interview: An ethnographic approach (pp. 53–68). Berg. 10.4324/9781003087014

[bibr65-10497323241241225] Rashid M. Caine V. Goez H. (2015). The encounters and challenges of ethnography as a methodology in health research. International Journal of Qualitative Methods, 14(5), Article 160940691562142. 10.1177/1609406915621421

[bibr66-10497323241241225] Rinaldo R. Guhin J. (2022). How and why interviews work: Ethnographic interviews and meso-level public culture. Sociological Methods & Research, 51(1), 34–67. 10.1177/0049124119882471

[bibr67-10497323241241225] Rosenberg D. (2001). Three steps to ethnography: A discussion of interdisciplinary contributions. AI & Society, 15(4), 295–315. 10.1007/BF01206112

[bibr69-10497323241241225] Roulston K. (2019). Epistemology and interviews. In Noblit G. W. (Ed.), Oxford research encyclopedia of education (pp. 18–19). Oxford University Press. 10.1093/acrefore/9780190264093.013.551

[bibr70-10497323241241225] Skågeby J. (2011). Online ethnographic methods: Towards a qualitative understanding of virtual community practices. In J. Behrendtz (Ed.), Handbook of research on methods and techniques for studying virtual communities: Paradigms and phenomena (pp. 410–428). IGI Global. 10.4018/978-1-60960-040-2.CH025

[bibr71-10497323241241225] Skinner J. (2012). A four-part introduction to the interview: Introducing the interview; society, sociology and the interview; anthropology and the interview; anthropology and the interview—Edited. In Skinner J. (Ed.), The interview: An ethnographic approach (pp. 1–50). Berg. 10.4324/9781003087014

[bibr102-10497323241241225] Smith L. T. (1999). Decolonizing methodologies: Research and indigenous peoples. Bloomsbury Publishing.

[bibr72-10497323241241225] Snodgrass J. G. (2016). Online virtual worlds as anthropological field sites: Ethnographic methods training via collaborative research of Internet gaming cultures. Annals of Anthropological Practice, 40(2), 134–147. 10.1111/napa.12097

[bibr73-10497323241241225] Song M. Parker D. (1995). Commonality, difference and the dynamics of disclosure in in-depth interviewing. Sociology, 29(2), 241–256. 10.1177/0038038595029002004

[bibr74-10497323241241225] Spradley J. P. (1979). The ethnographic interview. Waveland Press.

[bibr75-10497323241241225] Stacey J. (1988). Can there be a feminist ethnography? Women’s Studies International Forum, 11(1), 21–27. 10.1016/0277-5395(88

[bibr76-10497323241241225] Stage C. W. Mattson M. (2003). Ethnographic interviewing as contextualized conversations. In Clair R. P. (Ed.), Expressions of ethnography: Novel approaches to qualitative methods (pp. 97–106). SUNY Press.

[bibr77-10497323241241225] Tammivaara J. Enright D. S. (1986). But where is the hypothesis? A guide to reading and evaluating ethnographic studies. The Journal of Educational Foundations, (1), Article 106.

[bibr78-10497323241241225] Tedlock B. (1991). From participant observation to the observation of participation: The emergence of narrative ethnography. Journal of Anthropological Research, 47(1), 69–94. 10.1086/jar.47.1.3630581

[bibr79-10497323241241225] Thorne S. Kirkham S. R. O'Flynn-Magee K. (2004). The analytic challenge in interpretive description. International Journal of Qualitative Methods, 3(1), 1–11. 10.1177/160940690400300101

[bibr80-10497323241241225] Timmermans S. Tavory I. (2007). Advancing ethnographic research through grounded theory practice. In K. Charmaz, & A. Bryant (Eds.), Handbook of grounded theory (pp. 493–513). Sage. 10.4135/9781848607941

[bibr81-10497323241241225] Tong A. Sainsbury P. Craig J. (2007). Consolidated criteria for reporting qualitative research (COREQ): A 32-item checklist for interviews and focus groups. International Journal for Quality in Health Care, 19(6), 349–357. 10.1093/intqhc/mzm042 17872937

[bibr82-10497323241241225] Townsend R. C. Cushion C. J. (2021). ‘Put that in your fucking research’: Reflexivity, ethnography and disability sport coaching. Qualitative Research, 21(2), 251–267. 10.1177/1468794120931349

[bibr101-10497323241241225] Trundle C. Phillips T. (2023). Defining focused ethnography: Disciplinary boundary-work and the imagined divisions between ‘focused’ and ‘traditional’ ethnography in health research – A critical review. Social Science & Medicine, 332, Article 116108. 10.1016/j.socscimed.2023.116108 37531908

[bibr83-10497323241241225] Wacquant L. J. Bourdieu P. (1992). An invitation to reflexive sociology. University of Chicago Press.

[bibr84-10497323241241225] Walford G. (2007). Classification and framing of interviews in ethnographic interviewing. Ethnography and Education, 2(2), 145–157. 10.1080/17457820701350491

[bibr85-10497323241241225] Way E. (2013). Understanding research fatigue in the context of community-university relations [Master’s thesis, Clark University]. Local knowledge: Worcester Area Community-Based Research. https://commons.clarku.edu/localknowledge/3

[bibr86-10497323241241225] Westby C. Burda A. Mehta Z. (2003). Asking the right questions in the right ways: Strategies for ethnographic interviewing. The ASHA Leader, 8(8), 4–17. 10.1044/leader.ftr3.08082003.4

[bibr87-10497323241241225] Westby C. E. (1990). Ethnographic interviewing: Asking the right questions to the right people in the right ways. Journal of Childhool Communication Disorders, 13(1), 101–111. 10.1177/152574019001300111

[bibr88-10497323241241225] White K. Willis E. (2002). Positivism resurgent: The epistemological foundations of evidence-based medicine. Health Sociology Review, 11(1–2), 5–15. 10.5172/hesr.2002.11.1-2.5

[bibr89-10497323241241225] Willis A. Allen W. (2011). The importance of cultural humility in cross cultural research. International Journal of Innovative Interdisciplinary Research, 1(1), 111–119.

[bibr90-10497323241241225] Wright S. (1998). The politicization of ‘culture’. Anthropology Today, 14(1), 7–15. 10.2307/2783092

[bibr91-10497323241241225] Zhao P. Li P. (2023). The affordances of videoconferencing technology for doing interviews with children online: Methodological explorations based on a critical ethnography. Qualitative Inquiry, 29(10), 1033–1044. 10.1177/10778004231165464

